# Biomechanical Comparison of Different Fixation Methods for Treating Jones Fracture of the Fifth Metatarsal

**DOI:** 10.3390/bioengineering13020135

**Published:** 2026-01-23

**Authors:** Cheng-Min Shih, Yu-Chun Yen, Chun-Hsiang Wang, Yu-Heng Huang, Shun-Ping Wang, Kuo-Chih Su

**Affiliations:** 1 Department of Orthopedics, Taichung Veterans General Hospital, Taichung 407219, Taiwan; 10chengmin@gmail.com; 2 Department of Physical Therapy, Hungkuang University, Taichung 43304, Taiwan; 3 Department of Medical Research, Taichung Veterans General Hospital, Taichung 407219, Taiwan; yuchunyen@vghtc.gov.tw (Y.-C.Y.); wangch@vghtc.gov.tw (C.-H.W.); 4 Department of Post-Baccalaureate Medicine, College of Medicine, National Chung Hsing University, Taichung 402202, Taiwan; lodon2014@gmail.com; 5 Department of Medical Equipment Development and Application, Hungkuang University, Taichung 433304, Taiwan

**Keywords:** Jones fracture, implant, screw, plate, biomechanics

## Abstract

Jones fractures are Zone 2 fractures of the fifth metatarsal. Biomechanical comparisons of fixation strategies for Jones fractures remain limited by the lack of standardized, head-to-head evaluations across major fixation methods. The purpose of this study was to perform a standardized biomechanical comparison of six fixation configurations representing the three primary surgical techniques for Jones fractures and to examine the mechanical factors underlying differences in early construct stability. A synthetic fifth metatarsal model with a simulated Zone 2 fracture was stabilized using lateral plate fixation with different screw configurations, Kirschner wire fixation with or without tension-band wiring, or intramedullary headless screw fixation. All constructs were tested under displacement-controlled cantilever bending, and the force required to reach 1 mm of fracture site displacement was obtained and construct stiffness was calculated. Plate-based fixation demonstrated the highest resistance to bending deformation, followed by intramedullary screw fixation, whereas Kirschner wire-based constructs exhibited the lowest stability. These differences were explained by variations in load-sharing pathways and effective working length among fixation constructs. The addition of tension-band wiring did not result in a measurable improvement in stability compared with Kirschner wire fixation alone, consistent with the dependence of tension-band mechanisms on active muscle loading not represented in the experimental model. These findings provide a unified biomechanical comparison of commonly used fixation constructs for Jones fractures and clarify the mechanical basis for differences in early construct stability.

## 1. Introduction

A Jones fracture is a fracture at the metaphyseal–diaphyseal junction (zone 2) of the fifth metatarsal. It was first described by Sir Robert Jones in 1902. He sustained this injury while dancing, accidentally stepping on the lateral border of his foot [1,2].

Metatarsal fractures constitute a notable portion of foot injuries, accounting for approximately 35% of all foot fractures and 5–6% of all skeletal injuries [3]. In an adult population-based cohort from the United Kingdom, the mean age of patients with metatarsal fractures was 42 years, and fractures of the fifth metatarsal were the most common subtype [4]. Within fifth metatarsal fractures, Jones fractures are clinically important because they frequently affect active individuals and athletes, and prolonged time away from sport can be consequential [5].

Unlike a typical fracture caused by direct impact that leads to breakage of the bone, a Jones fracture is induced by indirect loading. It is typically induced by accidental inversion of the forefoot, with the body weight on the foot. The fracture site lies within a relative vascular “watershed” region between metaphyseal and diaphyseal arterial supplies, which has been implicated in impaired healing [6]. This vascular deficiency can contribute to poor healing or even nonunion at this site, making the treatment/management of a Jones fracture challenging [6,7].

Management strategies for Jones fractures are broadly categorized into conservative and surgical approaches. Conservative management typically includes a period of immobilization with restricted or non-weight-bearing, avoiding surgical morbidity but potentially prolonging time to union in higher-demand patients [3,5]. Surgical fixation aims to improve mechanical stability and facilitate earlier functional recovery but introduces risks such as wound complications, hardware irritation, and implant-related failure [2,5]. In a National Football League-specific review, refracture after operative treatment ranged from 4% to 12%, and incomplete healing ranged from 7% to as high as 50% across series [8]. However, there is a lack of consensus on which class of treatment is preferred. Novel/emerging surgical methods, such as combined modality approaches, have been reported [9]. Recent studies indicate that while surgery is often favored for athletes, nonoperative management can yield similar healing times in selected zone 2 fractures [10,11]. Thus, the optimal approach remains debated.

A compact critical review of the biomechanics literature reveals limitations in current knowledge. Unthan et al. compared cannulated screw osteosynthesis with tension-band wiring and reported construct-dependent differences in stability; however, the model was limited to specific constructs and did not address broader plate configurations or clinically relevant cyclic loading [12]. Su et al. compared multiple intramedullary screw constructs (including F.E.R.I. techniques and headless compression screws) in a synthetic model; the work clarified screw-construct effects but did not include extramedullary plate or wire constructs for cross-technique benchmarking [13]. Dobrotă et al. compared bicortical screw versus plate fixation using 3D-printed metatarsal models; although they reported stability differences between constructs, assumptions inherent to printed models and simplified boundary conditions limit direct translation to operative decision-making [14]. Collectively, these studies highlight the need for a standardized, head-to-head comparison that includes the major technique classes within a single protocol, using clinically interpretable stability metrics.

The purpose of the present study was to perform a biomechanical comparison of the aforementioned three fixation methods, with specific reference to stability of the fracture site. Specifically, we quantified construct stiffness (N/mm) and the force required to reach 1-mm displacement load (N) for intramedullary headless screw fixation, tension-band wiring, and stainless steel plate fixation using a synthetic fifth metatarsal model under controlled, non-destructive loading conditions, to characterize the relative biomechanical behavior of different fixation constructs during the early postoperative period.

## 2. Materials and Methods

### 2.1. Specimen Preparation

Our team utilized synthetic foot bones (SYNBONE model 9147; SYNBONE AG, Zizers, Switzerland) to simulate Jones fractures located at the proximal end of the 5th metatarsal. According to the manufacturer’s specifications, the synthetic foot bone model is constructed from solid foam material with a nominal density of approximately 0.35 g/cm^3^. This synthetic model was selected to provide a consistent fifth metatarsal geometry and a standardized testing substrate for comparative biomechanical evaluation of different fixation methods.

We conducted biomechanical stability testing using three different fixation methods to evaluate their effectiveness. Initially, we created fractures within Zone 2 of the proximal 5th metatarsal. After creating the fracture, we applied various fixation devices to stabilize the fractured bones. The distal sections of these stabilized fractured 5th metatarsal bones were then embedded in polyester resin. This embedding process ensured that the bones remained in a fixed position during subsequent testing, providing a consistent and stable environment for measurement.

The specimens were then secured using specialized fixtures designed to hold the bones firmly in place. These fixtures were crucial in maintaining the correct positioning of the specimens, preventing any movement that could affect the test results. Once secured, the specimens underwent biomechanical testing using a Materials Test System (MTS, model JSV-H1000, Japan Instrumentation System, Nara, Japan). The MTS machine applied controlled forces to the bones, simulating the mechanical loads they would experience in a real-life scenario.

### 2.2. Fracture Creation and Fixation

**Figure 1:**
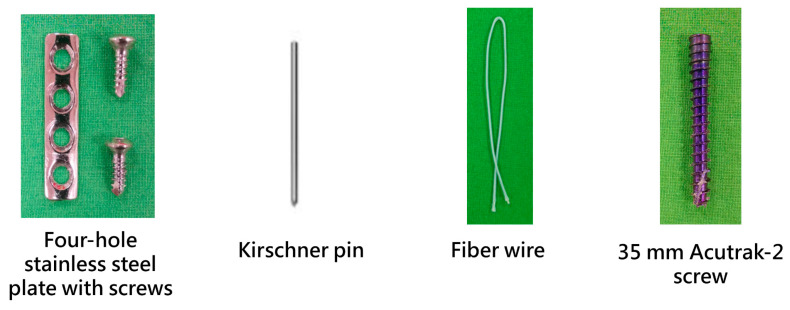
The bone plate, screws, Kirschner pin, fiber wire, and Acutrak-2 screw used in this study.

On a table, we marked 15 mm from the base of the 5th metatarsal and utilized an electric saw to create an oblique fracture at the metaphyseal–diaphyseal junction (Zone 2) of the fifth metatarsal. For the plate fixation groups, a 4-hole stainless steel bone plate was placed on the lateral side of the proximal metatarsal, centered over the fracture line, with two screw holes on either side of the fracture. The plate was secured using 2.7 mm cortical screws (Figure 1). For the Kirschner pins fixation group, two 1.6 mm Kirschner pins were inserted parallelly from the proximal metatarsal, passing through the fracture line, and exiting the cortical bone on the medial side of the distal metatarsal, achieving bicortical fixation. One subgroup used fiber wire (Arthrex, Naples, FL, USA) for tension-band wiring. We drilled a small hole through the bone 1 cm distal to the fracture line from top to bottom, passed the fiber wire through it, looped it in a figure-8 pattern around the exposed Kirschner pins, and then tightened and secured (Figure 1). The intramedullary screw fixation group utilized Acutrak-2 screws (Acumed, Beaverton, OR, USA) featuring a 4.7 mm trailing thread diameter and a length of 35 mm. These screws are designed with a taper, having a 4.5 mm leading thread diameter, and are constructed with a variable pitch in titanium. Figure 1 shows the plate used in this study, which was secured with two 2.7 mm cortical screws, a Kirschner pin, fiber wire, and an Acutrak-2 screw.

### 2.3. Experimental Groups

**Figure 2:**
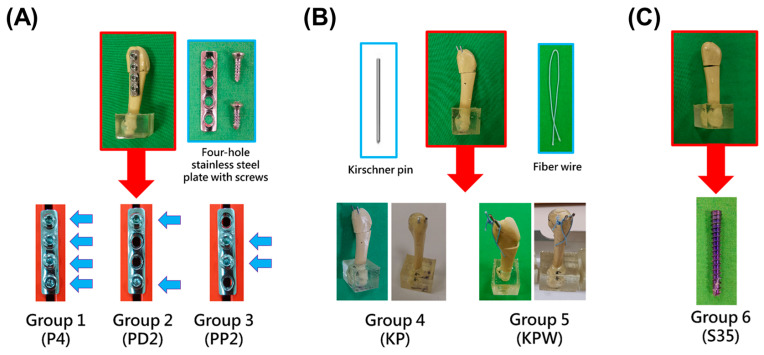
Illustration of the six experimental configurations evaluated in this study. The constructs were categorized into three fixation categories: (**A**) plate fixation, (**B**) Kirschner pins fixation, and (**C**) intramedullary screw fixation. In (**A**), plate fixation included three sub-groups using a four-hole stainless steel plate with different screw configurations: four screws (P4), two screws distal to the fracture line (PD2), and two screws proximal to the fracture line (PP2). Blue arrows indicate the positions of screw fixation on the plate. In (**B**), Kirschner pin fixation consisted of two parallel 1.6 mm Kirschner pins alone (KP) or combined with fiber wire for tension-band wiring (KPW). In (**C**), intramedullary fixation was performed using a 35 mm Acutrak-2 headless compression screw inserted from the proximal end of the fifth metatarsal (S35).

A total of six experimental groups were established to compare different configurations within the three most common fixation modalities (Figure 2). Plate fixation was used in three groups (Figure 2A): in the first sub-group, the plate was secured by four screws; in the second sub-group, the plate was secured by two screws distal to the fracture line; and in the third sub-group, the plate was secured by two screws proximal to the fracture line. Kirschner pins were used in two groups (Figure 2B): in the first sub-group, two Kirschner pins were used, and in the second sub-group, fiber wire was added. Intramedullary screw fixation was used in the third group (Figure 2C), with the screws inserted from the proximal end of the 5th metatarsal, passing through the fracture line until fully embedded, achieving intramedullary fixation. For each group, 10 tests were conducted, with displacement at the fracture ranging from 0.1 mm to 1 mm, in intervals of 0.1 mm.

The experimental groups evaluated in this study and their group labels are listed below.

• Group 1 (P4): Four-hole stainless steel plate with four screws.
• Group 2 (PD2): Four-hole stainless steel plate with two screws distal to the fracture.
• Group 3 (PP2): Four-hole stainless steel plate with two screws proximal to the fracture.
• Group 4 (KP): Two parallel 1.6 mm Kirschner pins for bicortical fixation.
• Group 5 (KPW): Two parallel 1.6 mm Kirschner pins with fiber wire for tension-band wiring.
• Group 6 (S35): Intramedullary fixation with a 35 mm Acutrak-2 screw.

### 2.4. Biomechanical Testing

In this study, the distal portion of the synthetic fifth metatarsal was embedded in polyester resin to provide rigid fixation during testing, while the proximal segment remained unsupported to create a cantilever configuration. All specimens were tested using a materials testing system (MTS; JSV-H1000, Japan Instrumentation System, Nara, Japan).

**Figure 3:**
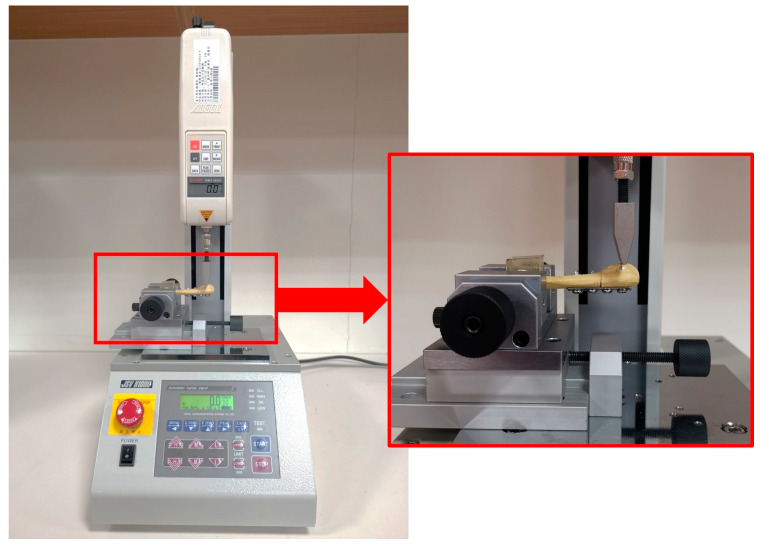
Experimental setup for biomechanical testing of the fifth metatarsal. The distal portion of the specimen was embedded in polyester resin, while the proximal segment remained unsupported to form a cantilever configuration. A dorsally directed bending load was applied using the MTS loading indenter positioned 10 mm from the base of the fifth metatarsal.

A displacement-controlled, dorsally directed bending load was applied to the proximal fifth metatarsal to evaluate the mechanical behavior of the fixation constructs. The loading indenter was positioned 10 mm from the base of the fifth metatarsal, and downward displacement was applied at this location to induce bending at the fracture site (Figure 3).

Prior to formal testing, a preloading protocol was performed to ensure consistent initial contact between the loading indenter and the bone surface. Using displacement control with a minimal step size of approximately 0.01 mm, the indenter was gradually advanced until a small initial contact force was detected by the load cell, indicating stable contact. At this point, displacement was stopped, and both force and displacement were zeroed to eliminate system slack.

Displacement was subsequently applied incrementally from 0.1 mm to 1.0 mm in 0.1 mm steps, and force-displacement data were recorded. For each specimen, the same loading protocol was repeated 10 times to assess measurement consistency.

This cantilever-based bending configuration represents a commonly used experimental setup in biomechanical studies for comparative evaluation of fixation methods [12].

At a displacement of 1.0 mm, the corresponding force was obtained from the load–displacement curve for each specimen. This force was defined as the 1-mm displacement load and was recorded to represent construct behavior under controlled, non-destructive deformation, without inducing fixation failure. Because excessive displacement at the fracture site is not expected immediately after surgery, the 1-mm displacement condition was used to examine the biomechanical stability of different fixation constructs in an early post-operative context. In addition, construct stiffness (N/mm) was calculated from the load–displacement relationship over the displacement range of 0.1 to 1.0 mm to characterize the overall mechanical rigidity of each fixation method.

### 2.5. Statistical Analysis

In this research, continuous data are presented as mean ± standard deviation (SD), whereas categorical data are expressed as frequencies and percentages. The normality of continuous variables was assessed using the Kolmogorov–Smirnov test. Group differences in the 1-mm displacement load and construct stiffness among the various fixation methods were analyzed using the Kruskal–Wallis test, followed by post hoc pairwise comparisons with Bonferroni correction. All statistical analyses were performed using IBM SPSS Statistics version 22.0 (International Business Machines Corp., New York, NY, USA). A *p*-value < 0.05 was considered statistically significant.

## 3. Results

**Figure 4:**
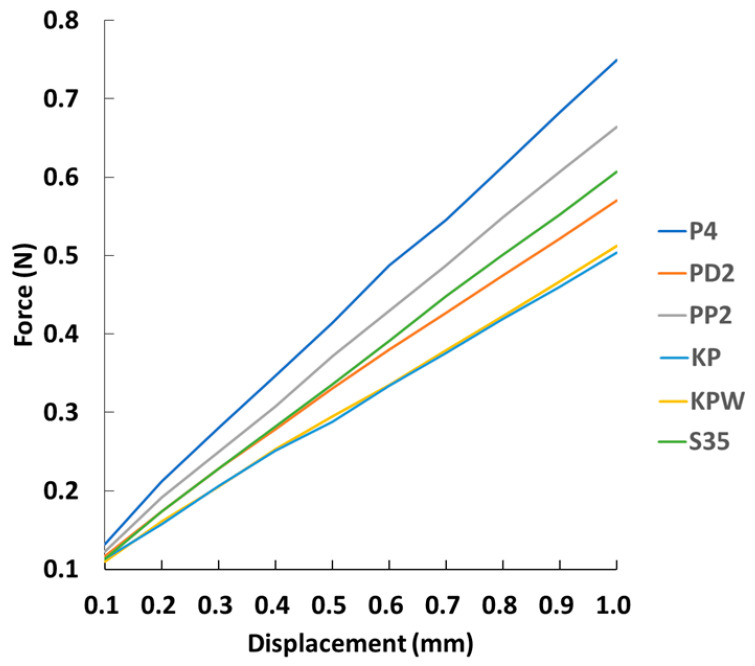
The relationship between force and displacement under different fixation methods.

Representative applied force–displacement results are presented in Figure 4. Distinct differences in mechanical behavior were observed among the fixation constructs over the displacement range tested. Plate-based fixation demonstrated consistently higher force resistance and steeper force–displacement slopes, whereas Kirschner wire-based constructs exhibited gentler slopes, indicating lower resistance to deformation across the same displacement range.

**Figure 5:**
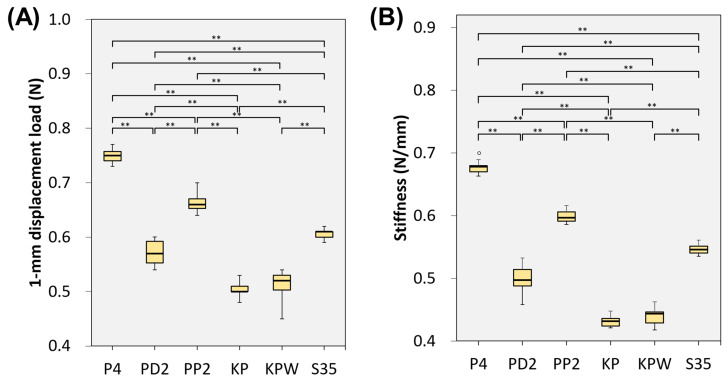
(**A**) 1-mm displacement load under different fixation methods. (**B**) Stiffness under different fixation methods. ** indicates statistically significant differences (*p* < 0.001).

Quantitative comparison at a displacement of 1.0 mm revealed significant differences in the 1-mm displacement load among fixation methods (Table 1, Figure 5A). The P4 construct exhibited the highest resistance to displacement (0.749 ± 0.011 N), followed by the PP2 (0.664 ± 0.016 N) and S35 (0.607 ± 0.009 N) constructs. In contrast, Kirschner wire-based constructs demonstrated substantially lower force values, with the KP (0.504 ± 0.005 N) and KPW (0.519 ± 0.015 N) groups showing no significant difference between them.

A similar trend was observed for construct stiffness (Table 1, Figure 5B). Plate-based constructs exhibited the highest stiffness, with the P4 group showing the greatest rigidity (0.676 ± 0.007 N/mm), followed by PP2 (0.598 ± 0.009 N/mm) and S35 (0.546 ± 0.008 N/mm). Kirschner wire-based constructs demonstrated the lowest stiffness values (KP: 0.431 ± 0.008 N/mm; KPW: 0.440 ± 0.014 N/mm), consistent with their gentler force–displacement slopes observed in Figure 4. The agreement between stiffness measurements and force–displacement behavior supports the observed differences in structural stability among fixation strategies.

## 4. Discussion

The purpose of this study was to directly compare the biomechanical stability of three commonly used surgical fixation strategies for Jones fractures (Zone 2) of the fifth metatarsal. Jones fractures occur in an anatomical watershed region with relatively limited blood supply and are clinically associated with higher risks of delayed union, nonunion, and refracture; therefore, the ability of a fixation construct to provide sufficient stability at the fracture site is of particular importance for high-demand patients who require early functional recovery and return to athletic activity [15]. Previous biomechanical studies have compared cannulated screws with tension-band wiring or have investigated the effects of different intramedullary screw designs, such as headless compression screws, on construct stability [12,13,16]. However, these studies were generally limited to comparisons of a small number of configurations, and substantial variability in model materials, loading conditions, and definitions of stability metrics (e.g., load–displacement criteria) has restricted meaningful cross-study comparisons among fixation techniques. At present, there remains a lack of head-to-head biomechanical evaluations conducted under a unified experimental framework that simultaneously includes intramedullary screw fixation, tension-band wiring, and lateral plate fixation as three major fixation categories [12,13,14,16]. To address this knowledge gap, the present study employed a standardized synthetic bone model and quantified construct stiffness and the force required to achieve 1 mm of fracture site displacement under consistent, non-destructive loading conditions. These results provide a biomechanical reference for cross-technique comparison and may serve as a mechanical basis for clinical consideration when evaluating stability requirements, implant selection, and postoperative weight-bearing or rehabilitation strategies.

Under the synthetic bone Jones fracture model and non-destructive bending loading conditions used in this study, all fixation constructs demonstrated consistent trends across both stability metrics, namely the force required to achieve 1.0 mm of displacement and construct stiffness. The P4 group exhibited the highest stability, followed by the PP2 construct, with S35 and PD2 showing intermediate performance, whereas the KP and KPW constructs demonstrated the lowest stability. Overall, intergroup differences were statistically significant (*p* < 0.001). In addition, no significant differences were observed between the KP and KPW groups, indicating that the addition of tension-band wiring did not result in a measurable improvement in construct stability under the present loading conditions. These differences can be explained by variations in load transfer pathways and effective working length among fixation constructs. The P4 construct provides multiple fixation points on both sides of the fracture, forming a relatively rigid lateral bridging configuration that allows externally applied bending loads to be efficiently distributed through the plate–screw system, thereby limiting fracture site displacement. Within plate-based constructs, the superior performance of PP2 compared with PD2 supports established mechanical principles: fixation points located closer to the fracture site shorten the effective working length and reduce cantilever effects, resulting in improved resistance to bending deformation [17]. Intramedullary screw fixation (S35) primarily controls displacement through internal support and thread–bone engagement, providing moderate stability [13]. In contrast, Kirschner wire-based constructs lack threaded fixation and exhibit limited bending rigidity, leading to the poorest stability performance [16]. From a mechanical perspective, the observed hierarchy in construct stability can therefore be attributed to fundamental differences in load-sharing mechanisms and resistance to bending deformation. Plate-based fixation spans the fracture site externally and distributes bending loads through multiple screw–bone interfaces, effectively shortening the working length and reducing bending moments. Intramedullary screw fixation relies on internal load sharing with a longer effective lever arm, while Kirschner wire constructs provide minimal resistance to bending under cantilever loading conditions. Notably, the addition of tension-band wiring did not result in a measurable improvement in construct stability when compared with Kirschner wire fixation alone. Although tension-band wiring is theoretically intended to convert tensile forces into compressive forces at the fracture site, this mechanism depends on the presence and direction of active muscle loading [18]. In the present study, loading was applied as a controlled, passive bending force and did not simulate the complex traction and rotational effects generated by the peroneus brevis muscle in vivo. Under these conditions, the tension-band construct is less able to generate effective fracture compression and therefore behaves mechanically in a manner similar to Kirschner wire fixation alone. This provides a biomechanical explanation for the lack of a significant difference between the KP and KPW groups observed in this study.

With respect to previously published biomechanical studies on fixation of Jones fractures, the relative trends observed in the present study are conceptually consistent with the existing literature, with plate-based constructs demonstrating the highest stability, intramedullary screw fixation showing intermediate stability, and Kirschner wire-based constructs exhibiting the lowest stability. Using synthetic bone models, Yılmaz et al. compared headless screws, tension-band wiring, and Kirschner wire fixation, and reported that Kirschner wire constructs showed significantly inferior stability and resistance to deformation compared with screw-based and tension-band systems. These findings indicate that K-wire-based fixation provides relatively limited resistance to displacement in this fracture region. At the same time, the relative biomechanical advantages of screw fixation and tension-band constructs were shown to depend on loading direction and testing configuration, and were not consistently observed across all experimental conditions, suggesting that the effectiveness of tension-band mechanisms is highly context dependent [16]. Under conditions of cyclic axial bending, Unthan et al. evaluated sixteen paired human cadaveric specimens and demonstrated no statistically significant difference in biomechanical stability between cannulated screw osteosynthesis and tension-band wiring [12]. In addition, substantial variability exists among prior studies with respect to model materials, including synthetic bone, cadaveric bone, and three-dimensional printed models, as well as loading modes such as bending, axial loading, and cyclic loading, and definitions of stability-related outcome measures. This heterogeneity limits direct numerical comparison across studies. For example, Dobrotă et al. used a standardized three-dimensional printed model to compare plate fixation with bicortical screw constructs and observed differences in resistance to deformation; however, differences in plate design and boundary conditions make these results more appropriate as conceptual support for the notion that plate fixation can increase overall construct stiffness under specific loading conditions, rather than as a direct quantitative comparison with the present findings [14]. Furthermore, Willegger et al. demonstrated that traction from the peroneus brevis tendon can serve as a source of postoperative instability following fixation of Jones fractures [18]. Because active muscle forces and complex rotational impulses were not incorporated into the experimental framework of the present study, the measurable biomechanical benefit of tension-band mechanisms may have been limited. This provides a plausible mechanical explanation for the absence of a significant difference between the KP and KPW groups observed in this study. Although plate fixation demonstrated higher resistance to displacement and greater construct stiffness in the present biomechanical model, this finding should not be interpreted as suggesting inferiority of intramedullary fixation or as a prescriptive recommendation regarding clinical treatment sequencing. Intramedullary fixation remains a widely accepted and effective treatment option for Jones fractures, offering well-recognized clinical advantages such as minimal surgical exposure, reduced soft tissue disruption, and favorable patient tolerance. Conversely, plate fixation, while providing greater structural stiffness in a controlled biomechanical setting, may be associated with potential drawbacks in clinical practice, including larger surgical incisions and implant-related irritation. Within this context, the present results are best understood as providing comparative biomechanical insight into the relative stability of different fixation constructs under identical loading conditions. The higher stiffness observed with plate fixation may be relevant in selected situations where enhanced initial mechanical stability is desired, but clinical decision-making should continue to balance mechanical considerations with biological, anatomical, and patient-specific factors.

Based on the above biomechanical comparisons, the force required to reach 1 mm of interfragmentary displacement and construct stiffness measured in the present study can be regarded as surrogate indicators of early postoperative fixation stability. From a mechanobiological perspective of fracture healing, construct stability influences interfragmentary motion and the local strain environment at the fracture site, which in turn affects callus formation and the progression of bone healing [19,20]. Accordingly, greater construct stiffness and higher resistance to displacement may, under equivalent external loading conditions, more effectively limit excessive motion and shear components, thereby providing a mechanical environment that is theoretically more favorable for fracture healing. However, recent reviews have emphasized that the relationship between implant stiffness and fracture healing is not strictly linear, and that clinical success requires a balance between sufficient stability to prevent deleterious displacement and allowance of controlled micromotion to promote healing. This balance may be influenced by factors such as fracture gap size, loading direction, and postoperative rehabilitation protocols [19,21]. With respect to clinical endpoints, studies on Jones fractures commonly use both clinical healing and radiographic healing as time-to-event outcomes. Therefore, more rigorous linkage of the biomechanical parameters measured in the present study to time to union, time to clinical healing, and time to radiographic healing would require validation based on established definitions and assessment criteria used in the foot and ankle literature [22]. It should also be noted that radiographic healing time is inherently influenced by the sensitivity of imaging modalities and interpretation criteria, and that conventional radiography has limited ability to detect early healing changes, potentially resulting in radiographic healing being identified later than the actual recovery of mechanical stability [23]. Finally, because the present study employed a synthetic bone model with quasi-static, non-destructive loading and did not incorporate cyclic fatigue loading or active muscle forces, the above clinical extrapolations should be regarded as reasonable postulates that warrant further verification using models that more closely replicate physiological conditions and through clinical follow-up studies.

The present study has several limitations related to the experimental model and loading conditions employed. A synthetic bone model was used to provide consistent geometry and material properties, enabling reproducible and direct comparison among fixation constructs. Although this model does not replicate all aspects of human bone behavior, it is suitable for identifying relative differences in construct stability under identical testing conditions. Fixation constructs were evaluated using a single-mode, displacement-controlled cantilever bending configuration. This simplified loading condition does not capture the full complexity of in vivo loading experienced by the fifth metatarsal, but it was intentionally selected to isolate construct-level mechanical behavior and to facilitate controlled comparison among fixation methods. In addition, testing was performed under quasi-static, non-destructive loading rather than cyclic or fatigue loading. This approach was designed to characterize early construct stability under limited displacement, which is relevant to the immediate postoperative setting. Accordingly, the findings represent relative structural stability under standardized bending conditions rather than direct estimates of long-term clinical performance.

This study provides a direct biomechanical comparison of six fixation variants derived from the three major surgical categories for Jones fractures under a unified experimental setting. While the present design focuses on early construct stability under controlled bending conditions, future work may extend these findings by exploring more complex loading scenarios or additional implant-related variables. Nevertheless, the current results offer a consistent biomechanical basis for understanding relative construct behavior and may serve as a useful reference when considering fixation stability in the early postoperative context.

## 5. Conclusions

This study performed a biomechanical comparison of six specific fixation configurations across three primary surgical techniques for Jones fractures of the fifth metatarsal. Plate-based fixation demonstrated greater resistance to bending deformation by spanning the fracture site externally and distributing loads through multiple fixation points, thereby shortening the effective working length and reducing bending moments. Intramedullary screw fixation provided intermediate stability through internal support and thread-to-bone engagement, whereas Kirschner wire-based constructs showed the lowest stability due to limited bending rigidity and the absence of threaded fixation. The lack of a measurable stability benefit with the addition of tension-band wiring highlights the context-dependent nature of this technique, as its ability to generate fracture compression depends on active muscle loading that was not represented in the present experimental model. Together, these findings explain the mechanical basis for the observed differences in early construct stability among fixation methods.

## Figures and Tables

**Table 1:** Mean ± standard deviation of the 1-mm displacement load and stiffness, derived from force–displacement curves recorded during displacement-controlled cantilever bending tests (0.1–1.0 mm displacement) for each fixation method. ** indicates statistically significant differences (*p* < 0.001).

	P4	PD2	PP2	KP	KPW	S35	*p*-Value
1-mm displacement load (N)							
mean ± standard deviation	0.749 ± 0.011	0.57 ± 0.022	0.664 ± 0.016	0.504 ± 0.005	0.519 ± 0.015	0.607 ± 0.009	<0.001 **
median	0.75	0.57	0.66	0.50	0.52	0.61	<0.001 **
Stiffness (N/mm)							
mean ± standard deviation	0.676 ± 0.007	0.499 ± 0.022	0.598 ± 0.009	0.431 ± 0.008	0.44 ± 0.014	0.546 ± 0.008	<0.001 **
median	0.678	0.497	0.596	0.432	0.443	0.546	<0.001 **

## Data Availability

The datasets presented in this article are not readily available due to institutional restrictions. Requests to access the datasets should be directed to the corresponding author.
